# Simplified regimen of combined low-dose rituximab for autoimmune encephalitis with neuronal surface antibodies

**DOI:** 10.1186/s12974-022-02622-8

**Published:** 2022-10-22

**Authors:** Ying Du, Chao Zhao, Juntong Liu, Chuan Li, Qi Yan, Lin Li, Yunfeng Hao, Dan Yao, Huaxing Si, Yingjun Zhao, Wei Zhang

**Affiliations:** 1 Department of Neurology, Tangdu Hospital, Fourth Military Medical University, Xi’an City, 710038 Shaanxi Province China; 2 Fujian Provincial Key Laboratory of Neurodegenerative Disease and Aging Research, Institute of Neuroscience, School of Medicine, Xiamen University, Xiamen, 361005 China

**Keywords:** Autoimmune encephalitis, Neuronal surface antibody, Rituximab, Low dose, Combined treatment, Clinical outcome

## Abstract

**Background:**

Autoimmune encephalitis (AE) with neuronal surface antibodies (NSAbs) presents pathogenesis mediated by B cell-secreting antibodies. Rituximab is a second-line choice for the treatment for AE with NSAbs, which can cause B cell depletion via targeting CD20. However, the optimal protocol and dosage of rituximab combined with first-line therapy for NSAbs-associated AE remains unclear so far. In this study, we explored the efficacy and safety of low-dose rituximab combined with first-line treatment for NSAbs-associated AE.

**Methods:**

Fifty-nine AE patients with NSAbs were enrolled, and retrospectively divided into common first-line therapy (41 patients) and combined low-dose rituximab (100 mg induction weekly with 3 circles, followed by 100 mg reinfusion every 6 months) with first-line therapy (18 patients). Outcome measures included changes in the Clinical Assessment Scale for Autoimmune Encephalitis (CASE) score (primary endpoint), changes in the modified Rankin Scale (mRS), the Mini-mental State Examination (MMSE), the patient and caregiver Neuropsychiatric Inventory (NPI) score at each visit (baseline, discharge, 6 months, 12 months and last follow-up) between two groups (secondary endpoint), as well as oral prednisone dosage, relapse and adverse effects during follow-up.

**Results:**

Compared with traditional first-line therapy group, for primary outcome, CASE scores at last follow-up were significantly improved in combined rituximab group, as well as markedly improving changes of CASE scores between baseline and each visit. While changes of mRS, MMSE and NPI scores, as secondary endpoint, were all markedly accelerating improvement between baseline and each visit, as well as both oral prednisone dosage and relapse were also greatly reduced during follow-up. Meanwhile, longitudinal analysis in combination of rituximab cohort also revealed persistently marked amelioration in a series of scales from baseline even more than 1 year. Moreover, analysis in rituximab subgroup showed no difference in any clinical outcomes between combination with single first-line and with repeated first-line treatment (≥ 2 times), while compared to delayed combination with rituximab (> 3 months), early initiation of combination (≤ 3 months) might achieve better improvements in CASE and MMSE assessment even 1 year later. No rituximab-correlated serious adverse events have been reported in our patients.

**Conclusions:**

Our simplified regimen of combined low-dose rituximab firstly showed significantly accelerating short-term recovery and long-term improvement for AE with NSAbs, in parallel with markedly reduced prednisone dosage and clinical relapses. Moreover, opportunity of protocol showed earlier initiation (≤ 3 months) with better long-term improvement.

**Supplementary Information:**

The online version contains supplementary material available at 10.1186/s12974-022-02622-8.

## Introduction

Autoimmune encephalitis (AE) is a new spectrum of immune-mediated disorders in central nervous system (CNS), characterized by pathogenic autoantibodies against neuronal surface or intracellular proteins [1]. Up to now, autoantibodies against neuronal proteins, especial neuronal surface antibodies (NSAbs), have been linked to more than 15 AE subtypes, such as anti-N-methyl-D-aspartate receptor (NMDAR)-AE, anti-leucine-rich glioma-inactivated-1 (LGI1)-AE and anti-contactin-associated protein-like-2 (CASPR2)-AE, making up the majority of seropositive AE subtypes [2]. Among them, most common NMDAR-AE, predominating in young women and children, mainly led to psychiatric symptoms, amnesia, epileptic seizures, reduced levels of consciousness and abnormal movements [3]. LGI1-AE, particularly affecting middle-aged or elderly patients, frequently caused confusion, short-term memory deficits, faciobrachial dystonic seizures and hyponatremia [4]. CASPR2-AE is mostly observed in elderly men, usually presented LGI1-like encephalitis and peripheral nerve hyperexcitability (neuromyotonia and myokymia) [5]. Despite heterogeneity of clinical features mediated by different antibodies in AE, the characterized manifestations were always involved in psychosis, cognitive impairments and seizures, far beyond motor dysfunctions [6]. NSAbs against surface antigens, such as NMDAR, LGI1 and CASPR2, may directly affect the targeted protein and cause clinical disturbances by blocking functions, interfering with synaptic protein interactions, or subsequent alterations of synaptic density. Furthermore, the underlying autoimmune processes may also lead to irreversible structural damages, as well as severe, progressive and refractory symptoms [7].

Treatment options for AE with NSAbs, ranging from broadly immunosuppressive agents to those targeting antibody-mediated pathogenesis, are mainly focused on achieving both better outcomes and fewer relapses. Common first-line immunotherapeutic agents for acute treatment include methylprednisolone pulse therapy (MPPT), intravenous immunoglobulin (IVIG), plasma exchange (PLEX) or combinations with less specificity for pathogenesis, presenting short-term efficacy during administration. The addition of a second-line agent such as rituximab, cyclophosphamide or combinations will be initiated, if there is no meaningful clinical or radiological response to optimized first-line treatment after 2–4 weeks [8]. Then, a steroid-sparing therapy or a bridging strategy of oral prednisone with a gradual taper overlapping with azathioprine or mycophenolate mofetil (MMF) after completing acute therapy, should be implemented for sustained immunosuppression [9]. There are no established guidelines for AE with NSAbs treatment so far, and the traditional protocol is often complicated and empirically performed according to patient status and clinician opinion. Given the balance between efficacy and safety, an aggressive and practicable approach of immunotherapy after definite diagnosis is critical for AE patients [10].

Recently, a retrospective study based on real-world data has indicated that high-dose rituximab (at least 1 g once) is the most frequent second-line immunosuppressive agent used in AE with NSAbs, but fails to present significant privilege in combination with first-line treatment because of more severity at baseline and complicated addition of drugs in the rituximab cohort, according to scores of modified Rankin Scale (mRS) [11]. Moreover, mRS, initially designed to measure motor function for stroke, has limitations for assessing non-motor deficits of AE mainly presenting psychosis, amnesia and seizures, thereby probably misleading objective evaluation of clinical outcomes. Therefore, a new specialized scale, named the Clinical Assessment Scale for Autoimmune Encephalitis (CASE), has been developed to rate the severity of AE comprehensively [12], and validated for AE with NSAbs in Chinese patients, presenting more sensitive to clinical changes than mRS [13, 14]. Meanwhile, up to now, the infusion regimen, optimal dosage and clinical benefit of combined rituximab for AE with NSAbs treatment still need to be elucidated. Here, we performed a retrospective study to assess the clinical outcomes for patients with NSAbs-associated AE, who were treated with common first-line medications only or with first-line medications and low-dose rituximab (an induction with 100 mg rituximab once a week for 3 cycles, followed by reinfusion 100 mg every 6 months at least for 1 year). We used improvement of CASE scores as the primary endpoint, and alterations in mRS, the Mini-mental State Examination (MMSE), the patient and caregiver Neuropsychiatric Inventory (NPI) scores as the secondary endpoints. We also evaluated clinical relapses, glucocorticoid reduction and adverse effects from different treatments. Without special notation, we will use the term “AE” to refer to AE with NSAbs only in the present study.

## Materials and methods

### Standard protocol approvals

This study was performed according to the Declaration of Helsinki, and approved by the Ethical Committee of Tangdu Hospital, Fourth Military Medical University. Moreover, we have provided patients and their relatives detailed information about the disease, and obtained the consent of the patients or their legal representatives to conventional first-line therapy and low-dose rituximab treatment, while written informed consent was obtained from all patients or their legal representatives.

### Study population

The study recruited 72 Chinese patients with NSAbs-associated AE diagnosed in the Department of Neurology of Tangdu Hospital from April 2015 to April 2021. Finally, 59 patients were retrospectively collected and analyzed, while the other 13 were excluded due to incomplete data or lost follow-up. Among them, 18 patients with combined low-dose rituximab met the following inclusion criteria: (1) patients with detection of NMDAR-, LGI1- or CASPR2- antibodies in CSF and/or serum, and definite diagnosis of NSAbs-associated AE according to published criteria [15]; (2) mRS scores ≥ 3 [16] or CASE scores ≥ 5 [13] at baseline screening; (3) any documented treatment with low-dose (100 mg once) rituximab, and available information on the number and timing of infusions; (4) patients with combined low-dose rituximab received prior first-line immune treatments, including MPPT 1000 mg daily for 5 days and/or IVIG 2 g/kg over 5 days (0.4 g/kg/day). The exclusion criteria were: (1) combination with other antibodies against neuronal and glia antigens; (2) disease complicated by potentially acute or chronic viral or bacterial infections, such as HIV, latent hepatitis B, tuberculosis, syphilis, viral encephalitis and so on; (3) presence of other severe neurological or psychiatric complications, such as brain tumor, stroke, myasthenia gravis and so on. In addition, a control cohort of total 41 patients with only first-line immunotherapy were also enrolled, with the consistent inclusion and exclusion criteria.

### Study design

All the patients had received at least one cycle of first-line immunotherapy defined as intravenous MPPT 1000 mg daily for 5 days, and/or IVIG 2 g/kg over 5 days (0.4 g/kg/day), followed by oral prednisone 30–60 mg/day for sustained immunosuppressive treatment with gradually tapering off [8, 9]. The combined regimen of rituximab was an induction of 100 mg once a week for 3 cycles, followed by reinfusions (100 mg once) at regular intervals (every 6 months) [17]. Relapse during follow-up was defined as new onset or worsening of encephalitis symptoms occurring after at least 2 months of improvement or stabilization [18], and judged by treating neurologists (Y Du, C Zhao and W Zhang) according to overall clinical impression. Simultaneously, detailed clinical status and lab examinations of each patient were evaluated at baseline and continuous 4 visits (discharge, 6 months, 12 months and last follow-up). Data on any immunotherapy and side effects of rituximab were recorded.

The primary efficacy endpoint of this study was the significant difference in the CASE score or accelerating improvement at each visit between combined low-dose rituximab treatment and common first-line therapy group. The secondary outcome measures were the marked differences in the mRS score, the MMSE score, the patient and caregiver NPI score or their accelerating improvements at each visit between two cohorts, as well as doses of oral prednisone and occurrence of relapses during the follow-up.

### Laboratory detection of neuronal surface antibodies

Antibody testing was performed by cell-based assays (CBAs) and confirmed by immunofluorescence (commercial test kit panels Euroimmun, Lübeck) for NMDAR, LGI1, and CASPR2. Patients reached the following inclusion antibody criteria: NMDAR antibody was detected in serum by CBA (> 1:100), followed by confirmation from immunofluorescence (in the absence of confirmatory immunofluorescence in serum, only CBA serum titers > 1:320 were considered specific) and/or CSF positive; LGI1 antibody at any titer in CSF and/or serum; CASPR2 antibody > 1:100 in serum and/or CSF positive [19]. Only IgG antibodies were considered relevant.

### Clinical assessment of immunotherapy profiles

As described previously, the CASE scale, evaluated for the current status of AE, consists of nine items, including seizure (current time), memory dysfunction, psychiatric symptoms (delusion, hallucination, disinhibition, aggression), consciousness, language problem, dyskinesia/dystonia, gait instability and ataxia, brainstem dysfunction, and weakness. The total maximum score was 27. Each item was based on a 3-point grading system, with the exception of the item “brainstem dysfunction”, which consisted of gaze paresis, tube feeding, and ventilator care due to hypoventilation [20]. Specifically, the items of “memory” and “language problem” were assessed mainly by communications and observations, rather than neurological examinations; the item of “seizure” was scored as 1 for controlled seizures with no need of dose-up, and scored as 2 for intractable seizures with the need of dose-up; in comatose patients, the items of “seizure”, “dyskinesia/dystonia”, and “brainstem dysfunction” could be used for evaluation, whereas all others were scored as 3 [13]. Moreover, two neurologists (J Liu and C Li) who were blinded to the diagnosis evaluated the scales independently by studying the detailed medical records, retrospectively. C Zhao repeated the assessment 1 month later.

The mRS scale consists of six grades (0–5 points), and predominantly captures the impact of motor deficits on functional independence [21]. The MMSE scale consists of 30 questions with the highest 30 points, and tests five cognitive domains, including time and place orientation (10 points), memory registration (3 points) and recall (3 points), attention and calculation (5 points), language and praxis (9 points), indicating higher scores with better cognition. Specifically, MMSE ≥ 27 is considered normal, 26 ≥ MMSE ≥ 21 is considered mild, 20 ≥ MMSE ≥ 10 is considered moderate, while MMSE ≤ 9 is considered severe cognitive impairment [22]. The NPI scale consists of 12 items of neuropsychiatric disturbances common in dementia, including delusions, hallucinations, agitation, dysphoria, anxiety, apathy, irritability, euphoria, disinhibition, aberrant motor behavior, night-time behavior disturbances, and appetite and eating abnormalities. The severity and frequency of each symptom are rated on the scripted questions for the patient’s caregiver, as well as assessment of caregiver distress by each neuropsychiatric disorder, then followed by a calculation of total NPI and total caregiver distress score [23]. All subjects were routinely assessed with mRS, MMSE, patient and caregiver NPI by two neurologists (D Yao and L Li) at baseline and each visit.

To adjust the possible differences in baseline functional status, the parameter “favorable clinical response” was analyzed at each visit under the following definition: improvement of the CASE scores by ≥ 5 points or achievement of the CASE scores ≤ 2 (as good) [20], improvement of the mRS scores by ≥ 2 points or achievement of the mRS scores ≤ 2 (as good) [21], or improvement of the MMSE scores by ≥ 10 points or achievement of the MMSE scores ≥ 27 (as good) [22].

### Statistical analyses

Statistical tests were performed using GraphPad Prism 8.0 (GraphPad Software, Inc., San Diego, CA) and SPSS version 26.0 (IBM, Armonk, NY, USA). Quantitative data with normal distributions were presented as mean ± SD. Continuous variables conformance to skew distributions such as CASE, mRS, MMSE, NPI and the difference of scale score before and after rituximab were described as medians with the interquartile range (IQR) and analyzed with a Wilcoxon signed-rank test and a Wilcoxon rank sum test. Symptoms and demographic data were analyzed using the *χ*^2^ test or Fisher exact test for categorical variables and Mann–Whitney *U* test for continuous variables. The time-weighted average prednisolone dose (mg/day) was calculated for each 4-week period after initial immunotherapy. The time-weighted average dose over each 4-week period was chosen to reflect inter and intraindividual variations in the interval and degree of dosage changes. One-way repeated measures analysis of variance (ANOVA) was conducted to analyze the effect of rituximab on the time-weighted average prednisolone dose. *p* < 0.05 was considered as significant.

## Results

### Patient characteristics with first-line or combined rituximab treatment

0According to the designed inclusion and exclusion criteria, we identified 59 patients (26 females, 33 males) with NSAbs-associated AE, including 41 NMDAR-AE, 12 LGI1-AE, and 6 CASPR2-AE patients. Among them, our study cohort comprised 18 patients in the combined rituximab cohort (14 with NMDAR-AE, 3 with LGI1-AE, and 1 with CASPR2-AE), 41 patients with only first-line therapy in the control cohort (27 with NMDAR-AE, 9 with LGI1-AE, and 5 with CASPR2-AE), and no patient suffered from tumor during study. Gender, age at onset, duration from onset to diagnosis, follow-up duration, CSF/MRI/EEG profiles, and CSF/serum antibody profiles showed no difference between two groups. The clinical symptoms of AE presented heterogeneous, including seizures, cognitive impairments, psychiatric symptoms, decreased consciousness, autonomic dysfunction, movement disorder and fever, and also showed no difference between two groups at baseline (Table 1).

**Table 1:** Characterization of the patient cohort

	Total			NMDAR-AE			LGI1-AE			CASPR2-AE		
	RTX (*n* = 18)	Ctrl (*n* = 41)	*p *value	RTX (*n* = 18)	Ctrl (*n* = 41)	*p *value	RTX (*n* = 18)	Ctrl (*n* = 41)	*p *value	RTX (*n* = 18)	Ctrl (*n* = 41)	*p *value
Gender; female/male	8/10	18/23	*0.505*	7/7	11/16	*0.406*	1/2	5/4	*0.100*	0/1	2/3	*–*
Age at onset, y; mean (95% Cl)	41.88 (33–50)	37.61 (32–42)	*0.356*	35.28 (27–42)	34.22 (28–40)	*0.823*	63.33 (48–78)	52.88 (43–62)	*0.190*	70 (–)	28.40 (19–37)	*–*
From onset to diagnosis, d; median (IQR)	20 (78)	24 (52)	*0.863*	14.5 (23.75)	16 (51)	*0.185*	35 (–)	60 (63)	*0.864*	190 (–)	31 (41)	*–*
Follow-up duration d; mean (95% Cl)	1058 (768–1349)	1362 (1136–1587)	*0.120*	1084 (802–1376)	1488 (1215–1556)	*0.059*	1086 (–)	1640 (1278)	*0.926*	244 (–)	561 (640)	*–*
Symptoms; *n* (%)												
Seizures	66.7%	61.0%	*0.455*	71.4%	66.7%	*0.100*	66.7%	55.6%	*0.100*	0.00%	40%	*–*
Cognitive impairment	94.4%	85.4%	*0.422*	92.9%	85.2%	*0.645*	100%	100%	*–*	100%	60%	*–*
Psychiatric symptoms	83.3%	87.8%	*0.690*	78.6%	88.9%	*0.393*	100%	88.9%	*0.100*	100%	80%	*–*
Decreased consciousness	44.4%	29.3%	*0.201*	57.1%	33.3%	*0.189*	0.0%	11.1%	*0.100*	0.0%	40%	*–*
Autonomic dysfunction	27.8%	22.0%	*0.742*	28.6%	25.9%	*0.100*	33.3%	0.0%	*0.250*	0.0%	40%	*–*
Movement disorder	44.4%	26.8%	*0.151*	50%	22.2%	*0.089*	33.3%	33.3%	*0.100*	0.0%	40%	*–*
Fever	44.4%	39.0%	*0.457*	57.1%	51.9%	*0.504*	0.0%	11.1%	*0.100*	0.0%	20%	*–*
CSF/MRI/EEG profiles												
CSF cc; median, (IQR)	7 (67)	8 (23)	*0.613*	8.5 (90.5)	12 (26)	*0.264*	4 (–)	2(4)	*0.282*	2 (–)	1 (62)	*–*
CSF protein; mean, (95% Cl)	404.45 (318–490)	353.82 (288–419)	*0.370*	372.05 (274–469)	405.57 (313–497)	*0.637*	470.28 (93–847)	247.21 (179–314)	***0.009***	660.7 (–)	266.32 (144–387)	*–*
CSF pressure; mean, (95% Cl)	174.72 (146–203)	161.46 (140–182)	*0.463*	182.85 (147–218)	170.18 (140–199)	*0.587*	145.00 (46–243)	131.66 (100–162)	*0.632*	150 (–)	168.00 (121–214)	*–*
MRI abnormalities; *n* (%)	50.0%	56.1%	*0.440*	64.3%	51.9%	*0.336*	0.0%	77.8%	***0.045***	0.0%	40%	*–*
EEG abnormalities; *n* (%)	61.1%	43.9%	*0.175*	71.4%	48.1%	*0.137*	33.3%	33.3%	*0.100*	0.0%	40%	*–*
CSF/serum Ab profiles												
CSF Ab positive	94.4%	80.5%	*0.252*	100%	88.9%	*0.539*	100%	77.8%	*0.100*	0.0%	40%	*–*
Serum Ab positive	38.9%	61.0%	*0.100*	28.6%	48.1%	*0.321*	66.7%	77.8%	*0.100*	100%	100%	*–*
Both Ab positive	33.3%	41.5%	*0.386*	28.6%	37.0%	*0.734*	66.7%	55.6%	*0.100*	0.0%	40%	*–*
Prior 1st-line immunotherapy; *n* (%)	100%	100%	*–*	100%	100%	*–*	100%	100%	*–*	100%	100%	*–*
1st-line therapy, *n* (%)												
MPPT	72.2%	97.6%	***0.008***	85.7%	93.3%	*0.265*	33.3%	100%	***0.045***	0	100%	*–*
IVIG	94.4%	46.3%	**<0*.0001***	92.9%	55.6%	*0.031*	100%	33.3%	*0.182*	100%	20%	*–*
Both	66.7%	43.9%	*0.092*	78.6%	51.9%	*0.176*	33.3%	33.3%	*0.100*	0	20%	*–*
RTX therapy												
Time from RTX therapy, d; median (IQR)	74.5 (410)	–	*–*	128.5 (405)	–	*–*	6 (–)	–	*–*	5 (–)	–	*–*
No. of RTX infusions, *n*; median (IQR)	5 (4)	–	*–*	5 (3)	–	*–*	3 (–)	–	*–*	3 (–)	–	*–*
Cumulative RTX dose, g; median (IQR)	500 (400)	–	*–*	500 (300)	–	*–*	300 (–)	–	*–*	300 (–)	–	*–*
1st to last infusion, d; median (IQR)	389 (864)	–	*–*	457.5 (863)	–	*–*	15 (–)	–	*–*	214 (–)	–	*–*
Averaged dose of prednisone, mg/day; median (IQR)	4.375 (7.67)	27.13 (21.67)	< 0*.0001*	5.563 (4.011)	28.89 (21.67)	<0*.0001*	0	10.83 (19.52)	*0.009*	0	–	*–*
Relapses; *n* (%)												
After 1st-line therapy	33.3%	12.2%	*0.074*	42.9%	18.5%	*0.140*	33.3%	0.0%	*0.250*	0	0	*–*
After RTX therapy	0	–	*–*	0	–	*–*	0	–	*–*	0	–	*–*

*AE* autoimmune encephalitis, *NMDAR* N-methyl-d-aspartate receptor, *LGI1* leucine-rich glioma-inactivated-1, *CASPR2* contactin-associated protein-like-2, *RTX* rituximab, *Ctrl* control, *Cl* confidence interval, *IQR* interquartile rang, *CSF* cerebrospinal fluid, *MRI* magnetic resonance imaging, *EEG* electroencephalogram, *cc* cell count, *Ab* antibody, *MPTP* methylprednisolone pulse therapy, *IVIG* intravenous immunoglobulin. *p* values reaching statistical significance are indicated in bold

Compared to control group with only first-line therapy, patients with combined low-dose rituximab received less MPPT (72.2% vs 97.6%, *p* = 0.008) but more IVIG (94.4% vs 46.3%, *p* < 0.0001), while no difference was observed for combination of both first-line treatments between two groups (66.7% vs 43.9%, *p* = 0.092). The combined regimen of low-dose rituximab for 18 patients was an induction of 100 mg rituximab once a week for 3 cycles, followed by reinfusions (100 mg once) at regular intervals (every 6 months). In total combined rituximab cohort, median time from rituximab therapy was 74.5 days, median number of rituximab infusions was 5 times, median dose of cumulative rituximab was 500 mg, and median time from initiation to last infusion was 389 days (Table 1).

### Clinical outcomes

**Fig. 1:**
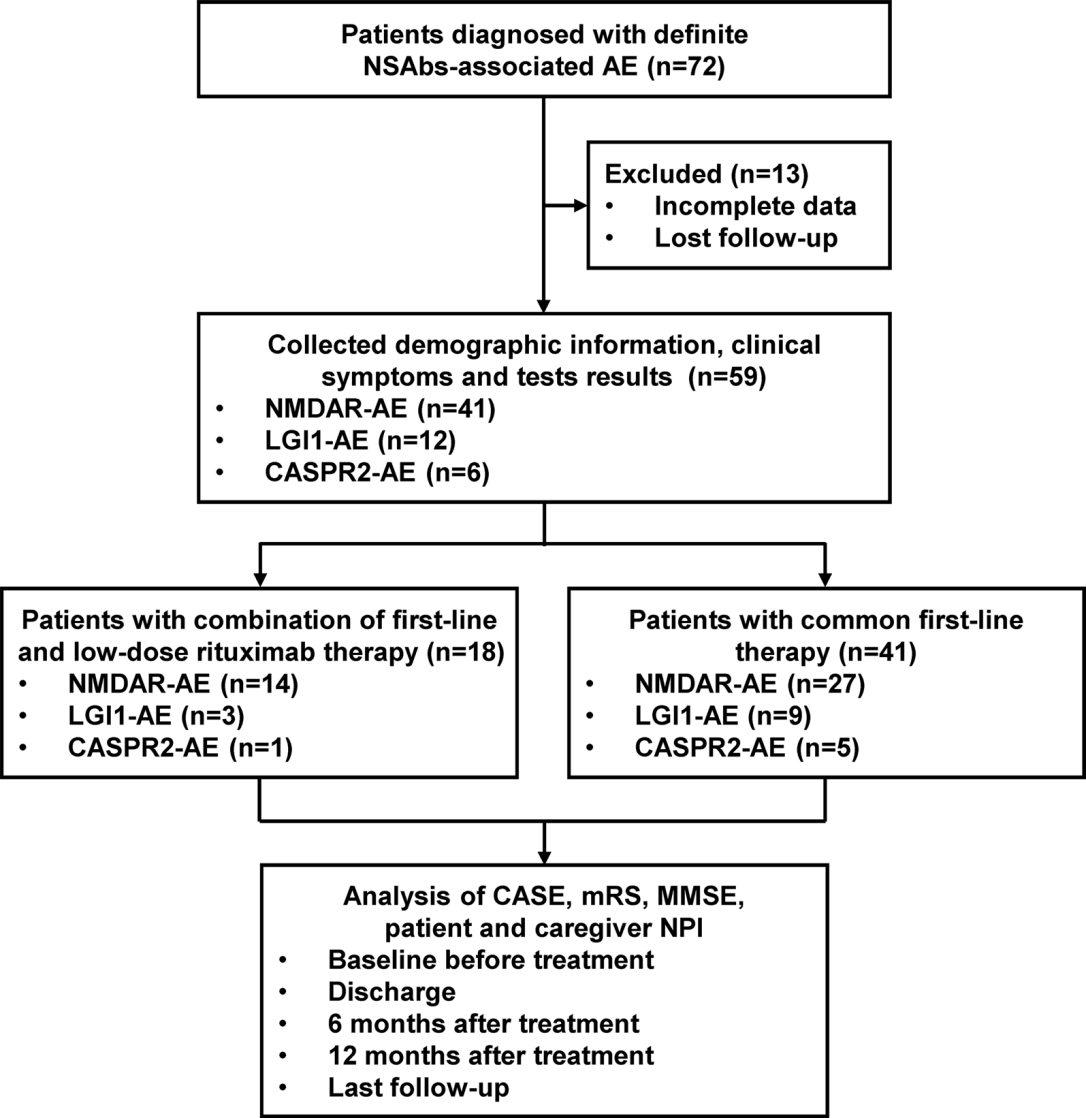
Flowchart of study design. NSAbs: neuronal surface antibodies; AE: autoimmune encephalitis, NMDAR: anti-N-methyl-d-aspartate receptor; LGI1 anti-leucine-rich glioma-inactivated 1; CASPR2: anti-contactin-associated protein-like-2; CASE: the Clinical Assessment Scale for Autoimmune Encephalitis, mRS: the modified Rankin scale, MMSE: the Mini-mental State Examination, NPI: the Neuropsychiatric Inventory

Compared to control cohort without rituximab, patients in combined rituximab cohort presented a more severe tendency in CASE, mRS, MMSE, patient or caregiver NPI score at baseline, but without significantly statistical differences (*p* > 0.05). However, at last follow-up, combined rituximab patients still showed a better outcome of CASE score than that of control group (*p* = 0.037). Moreover, in order to adjust the possible influence of baseline status, we also evaluated the improvement from baseline of CASE score at each visit, and the results still showed that patients with rituximab presented a significant improvement from baseline of CASE scores than those without rituximab at discharge (*p* = 0.01), 6 months (*p* = 0.016), 12 months (*p* = 0.013) and last follow-up (*p* = 0.001), respectively, suggesting the achievement of primary endpoint as designed in our study. Meanwhile, in the secondary outcome measures, clinical response reflected by a series of scales, including mRS, MMSE, patient and caregiver NPI, was noted at each visit. After adjusting the possible influence by baseline status, compared with control group, mRS, MMSE, patient and caregiver NPI also showed significant improvement from baseline in those with combined rituximab from discharge, and almost lasting till the study end, respectively (Table 2) (Fig. 1).

**Table 2:** Comparison of clinical outcomes between rituximab cohort and control cohort

Evaluation scales		Baseline			1st visit			2nd visit			3rd visit			Last visit		
		RTX	Ctrl	*p*	RTX	Ctrl	*p*	RTX	Ctrl	*p*	RTX	Ctrl	*p*	RTX	Ctrl	*p*
CASE	Scores median(IQR)	8 (7.25)	6 (2)	0.079	3 (4.5)	3 (2)	0.763	2 (3.25)	2 (2)	0.834	1.5 (1.25)	2 (2)	0.172	0 (1.25)	1 (2)	**0.037**
	Scores differ ± median (IQR)	–	–	–	− 5 (4.5)	− 3 (2)	**0.010**	− 6.5 (5.5)	− 3 (3)	**0.016**	− 6.5 (7.3)	− 4 (3)	**0.013**	− 7 (7)	− 4 (3)	**0.007**
mRS	Scores median(IQR)	4 (1.25)	4 (1)	0.083	2 (1)	3 (1)	0.270	2 (1)	2 (1.5)	0.771	1 (1.25)	1 (1)	0.532	0.5 (1.25)	1 (2)	0.418
	Scores differ ± median (IQR)	–	–	–	− 1 (1)	− 1 (0.5)	**0.010**	− 2 (10)	− 2 (2)	0.140	− 3 (2)	− 2 (1)	**0.043**	− 3.5 (2)	− 3 (1)	0.062
MMSE	Scores median (IQR)	14.5 (16.25)	20 (15.5)	0.062	23.5 (11)	23 (10)	0.785	25.5 (7.75)	24 (8)	0.907	26 (6.25)	27 (7)	0.522	28.5 (3)	27 (5)	0.089
	Scores differ ± median (IQR)	–	–	–	+ 8 (8.8)	+ 3 (7.5)	**0.006**	+ 11 (15.5)	+ 4 (13)	**0.046**	+ 10.5 (16)	+ 5 (14)	**0.037**	+ 13 (15.3)	+ 7 (14)	**0.018**
Patient NPI	Scores median (IQR)	15 (20.5)	9 (10)	0.066	1.5 (4.5)	3 (5.5)	0.532	0 (4)	0 (4)	0.863	0 (2)	0 (3)	0.538	0 (0)	0 (1.5)	0.371
	Scores differ ± median (IQR)	–	–	–	− 14 (14.3)	− 6 (10)	**0.021**	− 13 (16)	− 7 (11.5)	**0.044**	− 14 (20)	− 8 (11.5)	**0.038**	− 15 (20.8)	− 8 (11.5)	**0.028**
Caregiver NPI	Scores median (IQR)	6.5 (5.75)	5 (5)	0.054	1 (3)	2 (3)	0.461	0 (2)	0 (2)	0.785	0 (0.25)	0 (1.5)	0.381	0 (0.25)	0 (1.5)	0.547
	Scores differ ± median (IQR)	–	–	–	− 6 (5)	− 2 (4)	**0.006**	− 5 (7.3)	− 3 (5.5)	**0.043**	− 6.5 (7.8)	− 4 (5)	**0.038**	− 6.5 (6.3)	− 3 (5)	**0.016**

Detailed clinical status was evaluated by a series of AE-associated scales at baseline before treatment and continuous 4 visits after treatment. 1st visit: at discharge, 2nd visit: 6 months later, 3rd visit: 12 months later, 4th visit: last follow-up with at least > 12 months

*RTX* rituximab cohort, *Ctrl* control cohort, *CASE* the Clinical Assessment Scale for Autoimmune Encephalitis, *mRS* the modified Rankin Scale score, *MMSE* the Mini-mental State Examination score, *NPI* the Neuropsychiatric Inventory, *IQR* interquartile rang. *p* values reaching statistical significance are indicated in bold

**Fig. 2:**
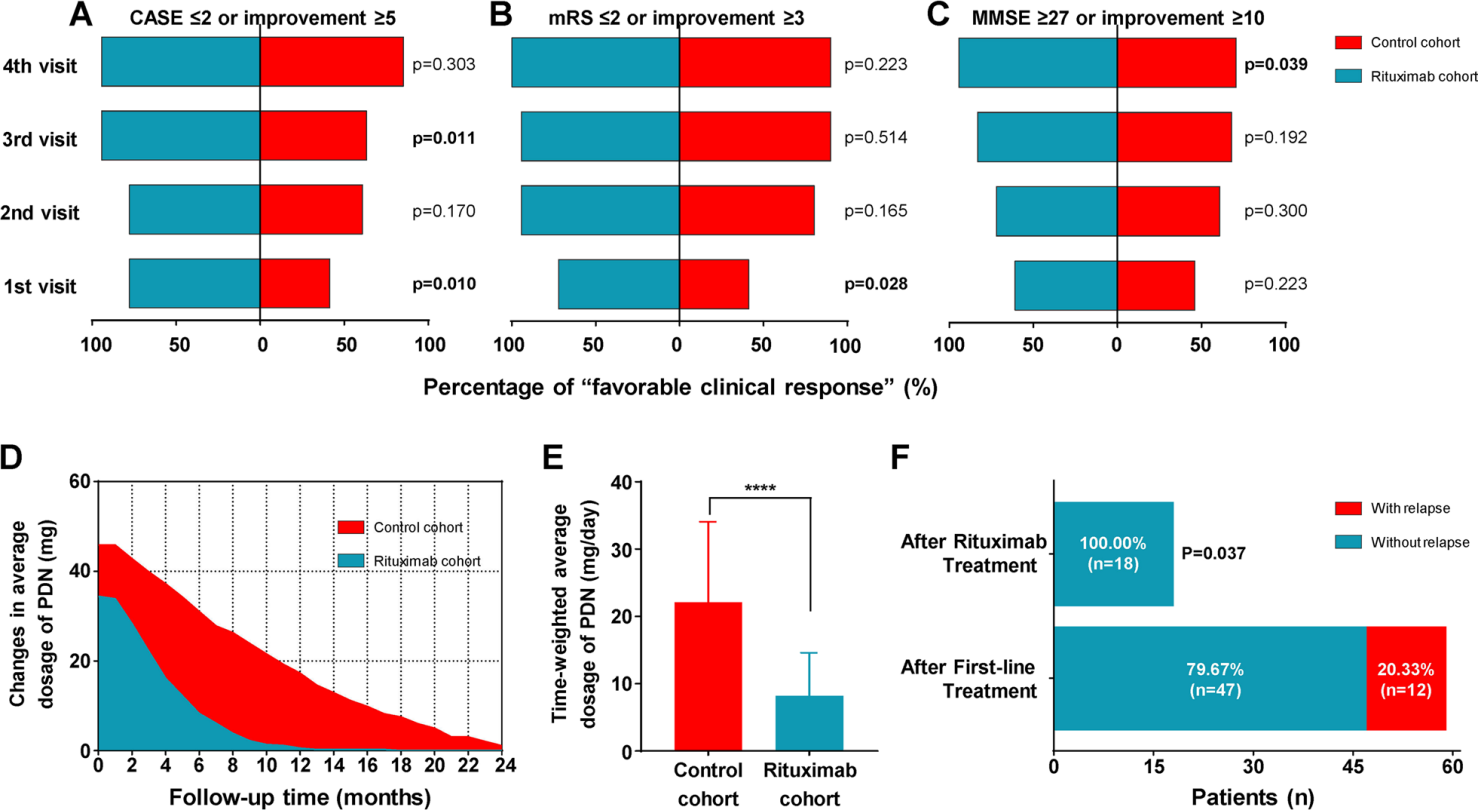
Comparison of clinical response, PDN dosage and relapse between control cohort and rituximab cohort. Detailed clinical status was evaluated by a series of AE-associated scales at baseline before treatment and continuous 4 visits (discharge, 6 months, 12 months and last follow-up) after treatment. Compared to control cohort with only first-line therapy, rituximab cohort with combination of first-line and rituximab therapy showed markedly higher percentage of “favorable clinical response” in three scales of AE, respectively, including CASE (**A**), mRS (**B**) and MMSE (**C**), as well as the cumulative oral dosage and time-weighted average dosage of prednisone were also significantly reduced in rituximab cohort (**D** and **E**). Moreover, compared with the percentage of relapse after total first-line treatment, it was also greatly decreased after rituximab treatment (**F**). In our original design, the “favorable clinical response” was defined as achievement of the CASE scores ≤ 2 or improvement of the CASE scores by ≥ 5 points, achievement of the mRS scores ≤ 2 or improvement of the mRS scores by ≥ 2 points, or achievement of the MMSE scores ≥ 27 or improvement of the MMSE scores by ≥ 10 points. CASE: the Clinical Assessment Scale for Autoimmune Encephalitis, mRS: the modified Rankin scale, MMSE: the Mini-mental State Examination, 1st visit: at discharge after treatment, 2nd visit: 6 months later, 3rd visit: 12 months later, 4th visit: last follow-up with at least > 12 months, PDN: prednisone. ****p < 0.0001

Furthermore, in the analysis of parameter for “favorable clinical response”, compared with control group, combined rituximab group presented better percentage in improvement of CASE scores at discharge (77.8% vs 41.5%, *p* = 0.01) and 12 months (94.4% vs 63.4%, *p* = 0.011) (Fig. 2A), as well as higher frequency in improvement of mRS scores at discharge (72.2% vs 41.5%, *p* = 0.028) (Fig. 2B) and MMSE scores at last follow-up (94.4% vs 70.7%, *p* = 0.039) (Fig. 2C). Meanwhile, compare to control cohort, the cumulative oral prednisone doses obviously decreased in rituximab cohort (*p* < 0.001) (Fig. 2D), as well as the time-weighted average prednisolone dosage in rituximab cohort was also markedly reduced than that in control cohort within the follow-up (*p* < 0.001) (Fig. 2E). Especially, during the study, 12 patients in total 59 patients had 12 relapses (X with NMDAR-AE, X with LGI1-AE). Among them, 7 relapses occurred before combined rituximab initiation in rituximab cohort (6 with NMDAR-AE, 1 with LGI1-AE), and no relapses occurred after rituximab treatment, while the other 5 relapses occurred in the control cohort (5 with NMDAR-AE). Cumulatively, there were 12 relapses after only first-line treatment in total AE patients, which was showed significantly frequent than that observed after initiation of rituximab (20.33% vs 0%, *p* = 0.037) (Fig. 2F).

### Treatment response and follow-up in each cohort

**Fig. 3:**
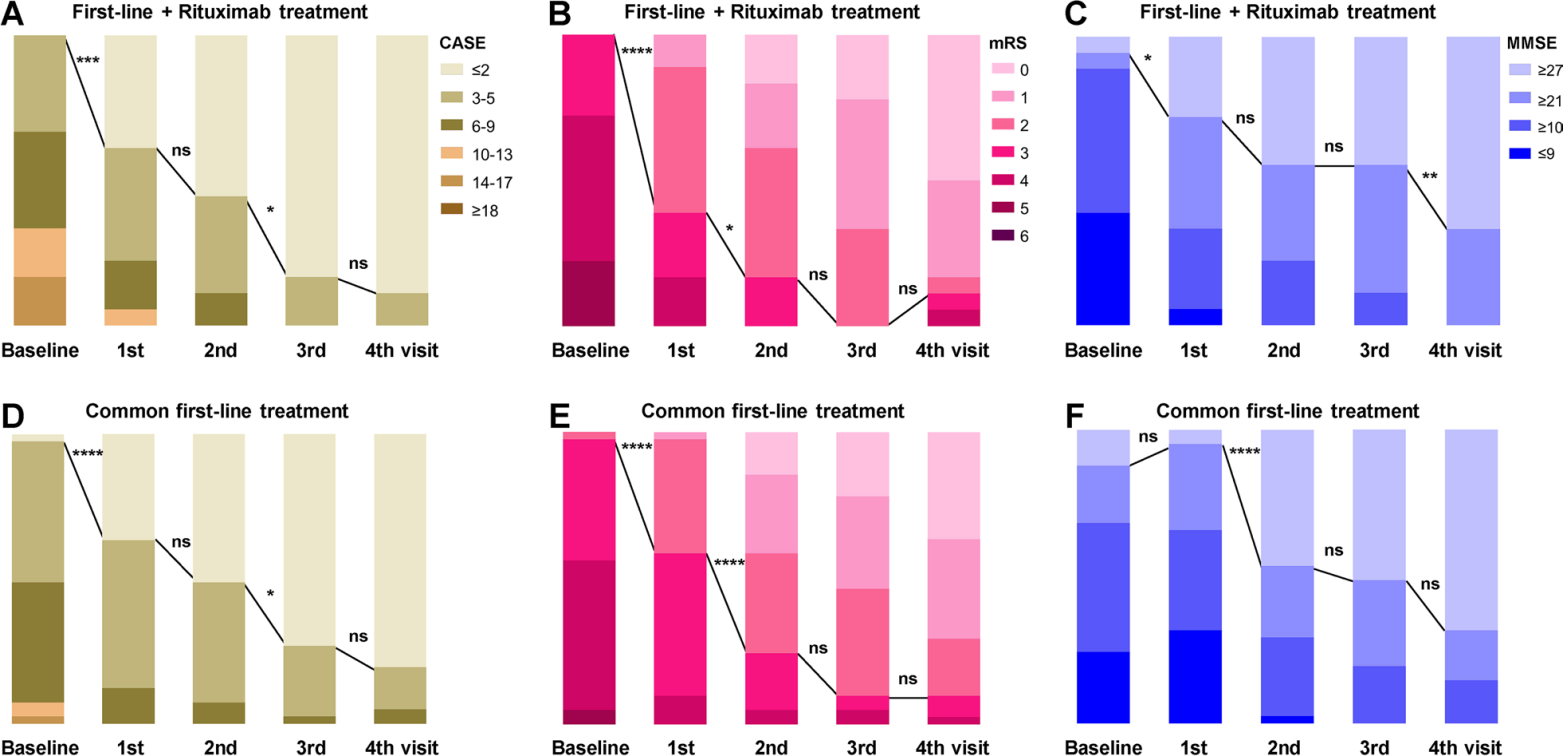
A series of AE-associated scales, including CASE, mRS and MMSE, was compared in the rituximab cohort with combination of first-line and rituximab treatment, and in the control cohort with only first-line treatment, respectively. The distribution of evaluation was depicted at 5 time points: baseline before treatment; 1st visit at discharge after treatment; 2nd visit at 6 months later; 3rd visit at 12 months later; 4th visit at last follow-up with at least > 12 months. The line represented the change in scores dividing “favorable clinical outcome” of CASE scores ≤ 2, mRS scores ≤ 2 or MMSE scores ≥ 27. The results were applied to compare the distribution of CASE (**A**), mRS (**B**) and MMSE (**C**) scores in rituximab cohort, as well as CASE (**D**), mRS (**E**) and MMSE (**F**) scores in control cohort. CASE: the Clinical Assessment Scale for Autoimmune Encephalitis, mRS: the modified Rankin scale, MMSE: the Mini-mental State Examination. ****p < 0.0001, ***p < 0.001, **p < 0.01 and *p < 0.05

Further analyses about each cohort, including control group with only first-line therapy and combined rituximab group, were also performed for longitudinal changes in CASE, mRS, MMSE, patient and caregiver NPI scores, respectively. In both control cohort and combined rituximab cohort, scores of CASE, mRS, MMSE, patient and caregiver NPI were all greatly improved from baseline at each visit (Additional file 1: Table S1). When we analyzed the CASE (≤ 2), mRS (≤ 2) or MMSE (≥ 27) scores for favorable outcomes in the rituximab cohort throughout follow-up in more detail, we found that patients with rituximab showed significant improvement in CASE scores at discharge (*p* = 0.001) and 12 months (*p* = 0.049) (Fig. 3A), continuously improving trend in mRS scores with achievement of statistical significance at discharge (*p* < 0.0001) and 6 months (*p* = 0.015) (Fig. 3B), and sustained improving trend in MMSE scores with achievement of statistical significance at discharge (*p* = 0.015) and last visit (> 12 months) (*p* = 0.004) (Fig. 3C). While in analysis of a series of scales in the control cohort without rituximab during follow-up, we also found that patients in control group presented significant improvement in CASE scores at discharge (p < 0.0001) and 12 months (*p* = 0.039), great amelioration in mRS scores at discharge (*p* < 0.0001) and 6 months (*p* < 0.0001), and marked promotion in MMSE scores only at 6 months (*p* < 0.0001). Altogether, these results proposed that both only first-line and combined rituximab therapy showed significantly comprehensive efficiency within 1 year by a series of scales assessment, while combined rituximab treatment seemed to especially present sustained improvement in cognitive impairment even 1 year later.

### Clinical analysis for subgroup in rituximab cohort

**Fig. 4:**
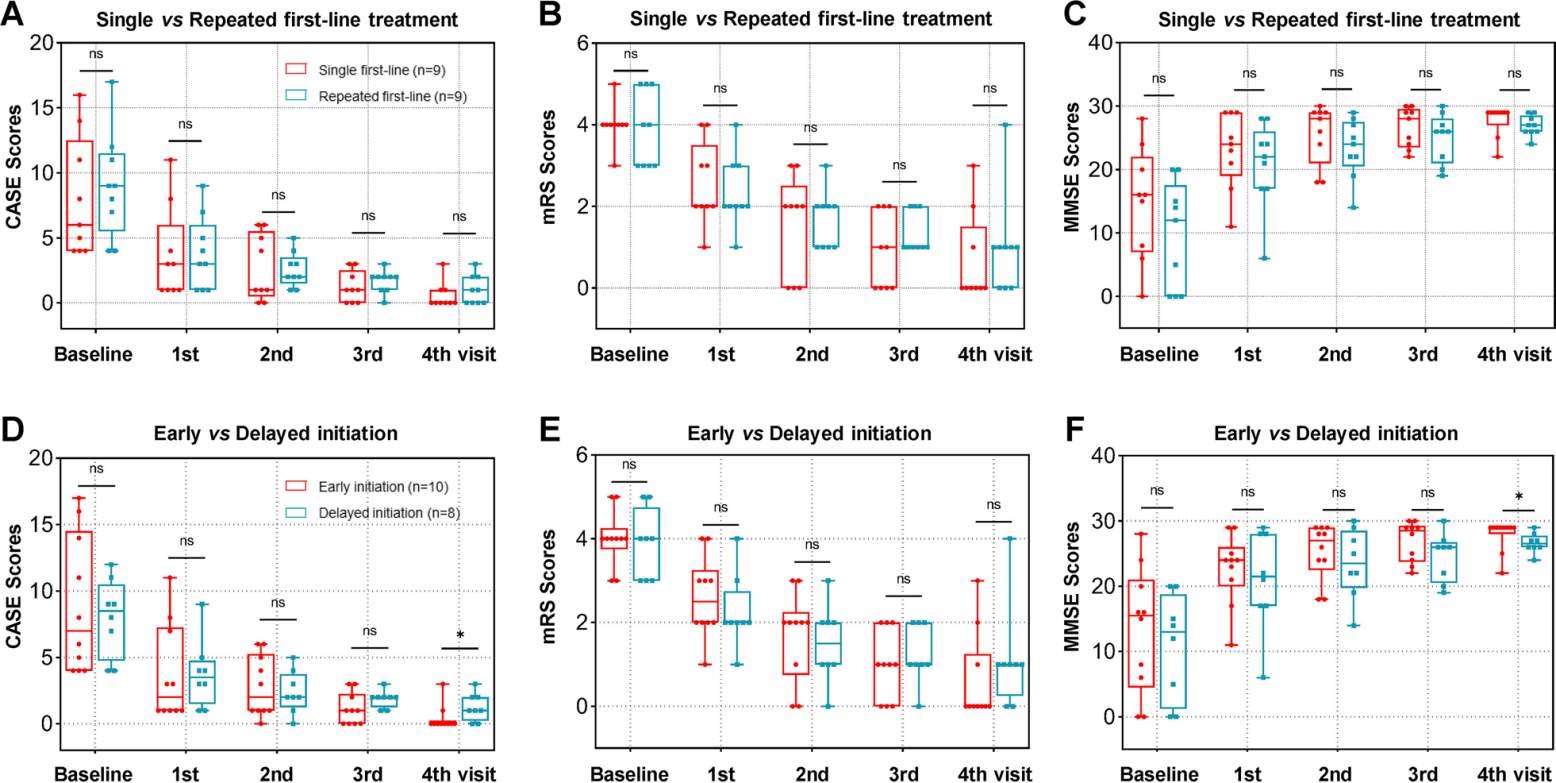
Effects of repeated first-line treatment and rituximab initiating opportunity on clinical outcome in different subgroups of rituximab cohort. Further analysis showed that, compared to single first-line treatment combined with rituximab subgroup, repeated first-line combined with rituximab presented no significant difference in CASE (**A**), mRS (**B**) or MMSE (**C**) evaluation at baseline and each visit. While compared to the subgroup with delayed initiation of rituximab over 3 months, early initiation of rituximab within 3 months led to a marked improvement in CASE (**D**) and MMSE (**F**) assessment at last follow-up, respectively, but still showed no significance in mRS scores (**E**) at baseline and each visit. CASE: the Clinical Assessment Scale for Autoimmune Encephalitis, mRS: the modified Rankin scale, MMSE: the Mini-mental State Examination, 1st visit: at discharge after treatment, 2nd visit: 6 months later, 3rd visit: 12 months later, 4th visit: last follow-up with at least > 12 months. *p < 0.05

In our study, all the 18 patients with rituximab received at least one cycle of prior first-line immunotherapy, defined as intravenous methylprednisolone 1000 mg daily for 5 days, and/or IVIG 2 g/kg over 5 days (0.4 g/kg/day). Among them, 9 patients were administrated first-line treatment only for one cycle, while the other 9 patients received repeated first-line therapy more than twice. Moreover, 10 patients in rituximab cohort were initiated rituximab within 3 months, while the other 8 patients were delayed for initiation over 3 months. Further analysis was performed to assess the possible influence of repeated first-line treatment and rituximab initiating opportunity on clinical outcome in different subgroups of rituximab cohort (Additional file 2: Table S2 and Additional file 3: Table S3). The results showed clearly that compared to single first-line treatment combined with low-dose rituximab subgroup, repeated first-line administration combined with rituximab presented no significant difference in CASE (Fig. 4A), mRS (Fig. 4B), MMSE (Fig. 4C), patient or caregiver NPI evaluation at baseline and each visit (Additional file 2: Table S2). However, compared to the subgroup with delayed initiation of rituximab over 3 months, early initiation of rituximab within 3 months led to a significant improvement in CASE (*p* = 0.039) (Fig. 4D) and MMSE (*p* = 0.046) (Fig. 4F) assessment at last follow-up, respectively, but still showed no significance in mRS scores (Fig. 4E), patient or caregiver NPI at baseline and each visit (Additional file 3: Table S3). Altogether, these results suggested that repeated first-line treatment presented no influence on clinical outcomes in patients with combination of rituximab, while early initiation of combined rituximab within 3 months seemed to showed significant advantages in long-term clinical improvement.

### Adverse effects and safety of rituximab

In a total of 18 patients on rituximab, two had infusion-related symptom which presented as skin rash, fever during the administration of rituximab. However, the symptoms gradually disappeared after oral cetirizine. Severe infusion adverse events did not occur in all patients. Of note, the side effects of glucocorticoids as central obesity, dropsy, acne, osteoporosis, abnormal glucose tolerance, hypertension, psychiatric disorder were often presented in non-rituximab cohort for long-term large oral doses of hormones.

## Discussion

In present study, the 3 main findings about rituximab treatment for AE are that: (1) our simplified regimen of low-dose rituximab (100 mg once) combined with common first-line therapy significantly accelerates comprehensive short-term recovery within 1 year, as well as markedly contributing to long-term improvement even after at least 1 year. (2) Our refined protocol of rituximab infusions leads to faster oral prednisolone gradual taper and withdrawal, in parallel with markedly sustained clinical remission and reduced relapses. (3) Opportunity of rituximab schedule shows earlier initiation with better improvement, while frequency of first-line therapy has no influence on satisfactory outcome with rituximab combination.

As we know, self-reactive B cells are subject to the processes of negative selection for elimination, such as deletion, receptor editing and induction of anergy, throughout the development in the bone marrow and spleen [24]. However, emerging data suggest defective B cell tolerance checkpoints in several AE (such as NMDAR-AE, LGI1-AE and CASPR2-AE), increasing autoreactive immature B cells that are not removed but can be activated and enter germinal centers [25, 26]. Several mechanisms may contribute to the loss of B cell tolerance in peripheral lymph nodes in the context of tumor ectopic expressions or potential viral infections, particularly by inducing B cell-intrinsic Toll-like receptor (TLR) signal together with B cell receptor (BCR) ligation, or activating T helper (Th) cell with same antigen stimulation, thereby leading to consecutive B cell clonal expansion, class switch, affinity maturation and NSAbs production [24, 27, 28]. Subsequently, activation of innate immune-mediated cytokines and TLR ligands leads to disruption of the blood brain barrier (BBB), allowing infiltration of autoreactive memory B and plasma cell, followed by proliferation with B cell activating factor (BAFF), and releasing large amounts of NSAbs in CNS [29]. Among them, anti-NMDAR antibodies are mainly IgG1 subclass, leading to rapid reduction of neuronal surface NMDAR by cross-linking, internalization and degradation, while antibodies against LGI1 and Caspr2 are predominantly IgG4, inducing neuronal dysfunction by interrupting the trans-synaptic binding of LGI1 or Caspr2 to its postsynaptic receptor a disintegrin and metalloprotease 22 (ADAM22) and likewise ADAM23 at the presynaptic site, thus causing a series of clinical phenotypes [30, 31].

Rituximab exerts therapeutic effect through its target, cluster of differentiation 20 (CD20), an integral membrane protein mainly expressed on B lymphocytes. During the autoimmune pathological process, the expression of CD20 is progressively increased in B cells at different developmental stages and sustainedly expressed at a high level on the surface of antibody-specific memory B cells and plasmablasts [32]. In NSAbs-associated AE, CD20 may act as a physically coupled link to BCR and other surface molecules or cytoplasmic proteins, such as major histocompatibility complex class II (MHCII), CD40 molecules, and tyrosine kinases, thereby regulating cell cycle progression and proliferation of B lymphocytes [33]. Moreover, activated CD20 + B cells can present same specific antigens to T lymphocytes in association with MHC molecules in the presence of various costimulatory factors, thus promoting T cells activation and differentiation. Subsequently, these T cells can produce a variety of cytokines and chemokines to regulate the maturation and migration of peripheral immune effectors, such as Th, CD8 + T and myeloid cells, secreting a range of proinflammatory mediators to induce neuroinflammation within the CNS parenchyma [34]. Therefore, rituximab may inhibit neuroinflammation via targeting CD20 + B lymphocytes, which results in beneficial effects for AE treatment.

Although instances of spontaneous recovery without immunotherapy have also been reported occasionally in AE, the disease is mostly presented as a progressively monophasic process with rare recurrence (approximately 10–20%) but apparent sequelae, suggesting the irreversible neuronal damages, and advocating the necessity for prompt and persistent interruption of pathogenic immune activity [35]. Traditional AE first-line therapy, including corticosteroids, IVIg, and PLEX, has limitation of less specificity for pathogenesis or shorter maintenance for treatment, while oral prednisone for bridging and steroid-sparing, azathioprine or MMF for sustained immunosuppression, both have common deficiency of less specificity, as well as complicated diverse regimen and continuous adverse effects [8, 9]. Proposals derived from recent systematic review for AE treatment are favored in early initiation of immunotherapy and addition of second-line agents, thereby resulting in better functional outcomes and lower relapses with manageable side effects [36]. Given the largely B cell-secreted antibody mediating the disease pathogenesis, it should be considered that a combination of immunotherapeutic agents targeting B cells may be urgently required for a more efficient regimen of AE treatment [37]. Therefore, rituximab is preferentially selected in second-line agents due to substantially special efficacy and relatively reliable safety.

Rituximab as a second-line agent for AE, initially approved for treatment of lymphoma, is a human/murine chimeric monoclonal antibody directed against a differentiation glycoprotein CD20 participating in B lymphocytes activation and proliferation. After binding to CD20 on the B cells surface, rituximab specifically depletes target B lymphocytes (such as naïve B cells, memory B cells and some plasmablasts) by antibody-mediated cellular toxicity, complement activation and induction of apoptosis, thereby reducing B cells response, and causing therapeutic immunosuppression [38]. Currently, activation of self-reactive B cells and their subsequent proliferation and differentiation into auto-antigen reactive memory B cells and autoantibody-secreting plasma cells, play pivotal pathogenic roles in antibody-mediated neurological diseases, such as AE, neuromyelitis optica spectrum disorders (NMOSD) and myasthenia gravis (MG). Therefore, the off-label use of rituximab for deleting the antigen-specific memory B-cell populations and hence preventing the formation of new plasmablasts which secrete the pathogenic antibodies, was gradually emphasized and presented potential advantages in AE treatment [34]. Consensus criteria on the appropriate time to initiate a second-line agent such as rituximab are yet to be established in AE, but a quick procession is favored, regardless of the response to first-line therapy. When rituximab is used since the acute setting, it may have the added benefit of a potentially faster onset of action, and also serve as a bridging therapy to prevent early relapses that might happen if immunosuppression is abruptly discontinued, as well as an optimal alternative for a sustained immunosuppressant [18]. Thus, the previously complex regimen for AE might be simplified and refined as combination of regular rituximab infusions with conventional first-line therapy.

In reference to a variety of researches about rituximab treatment in AE, there is great heterogeneity concerning dosages. Currently, the empirical protocols for AE are mainly derived from other disease processes such as lymphoma or rheumatoid arthritis (RA), including 375 mg/m^2^ weekly for 4 consecutive weeks or two doses of 1000 mg 2 weeks apart, then followed by reinfusions at fixed intervals for immunosuppressive maintenance, because of circulating B cell below the detectable range for 6–8 months after administration [39]. Moreover, the optimal dosage of rituximab for balancing between safety and efficacy are still ambiguous in AE treatment, and empirical off-label attempt primarily comes from high-dose therapy for lymphomas, usually exerting more medical expenses and serious adverse events [40]. Indeed, the dysfunctional B cells commonly present with normal circulating count in autoimmune diseases, which is different from the high tumor burden in lymphomas, and low-dose rituximab seems to be sufficient and effective for complete depletion of peripheral CD20 + B cells [41]. Recently, reduced low-dose of 100 mg rituximab per infusion for treatment has been tried in some neurological autoimmune disorders, such as NMOSD, MG, multiple sclerosis (MS) and neuro-Behçet’s disease (NBD), with the protocol of induction per week for 3 cycles, then followed by reinfusions at regular intervals. The approach still presented good responsive in depleting B cells, improving clinical symptoms and preventing relapses with favorable side-effect and medical cost [16, 42–44]. Thus, in present study, we performed a simplified regimen in AE treatment, including regular induction of 3 cycles for acute and bridging management, and subsequent reinfusions (100 mg once) at fixed interval (every 6 months) for sustained immunosuppression.

Meanwhile, the scales for evaluating clinical severity and therapeutic response in AE were also continuously improving and updating. Currently, because of no special tools for AE assessment, the modified Rankin scale (mRS) was widely applied to measure neurological severity and outcome [18, 20]. As we know, the mRS was primarily developed and mainly weighted for estimating prognosis of motor function in acute stroke management, while patients with AE usually presented a variety of symptoms beyond motor deficits, including behavioral changes, memory impairments, seizures, speech disorders, abnormal movements, decreased consciousness, and cerebellar ataxia, which might also interfere with each other in evaluation [12, 45]. Some other scales for special functional domains, such as MMSE for cognitive damages and NPI for neuropsychiatric symptoms, still had obvious limitations of lack diversity in assessment for AE ^[22. 23]^. Recently, prognostication and estimation tools specifically developed for AE, such as CASE scale with description in detail and validation in practice, might help to select those patients required for more aggressive immunotherapy, and comprehensively and accurately evaluate their clinical outcomes [12]. Hence, according to severity designed previously as mRS scores ≥ 3 or CASE scores ≥ 5, we retrospectively chose moderate and severe AE patients received first-line therapy with or without combination of low-dose rituximab in this study, and also discussed changes of CASE, mRS, MMSE, patient and caregiver NPI scale as well as glucocorticoid dosage and relapses between different cohorts.

As we found during the follow-up, compared to control cohort with common first-line therapy for AE, combined low-dose rituximab cohort not only showed much better outcome in CASE scale evaluation even 1 year later, but also significantly accelerated improvement in CASE, mRS, MMSE, patient and caregiver NPI from baseline within 1 year, as well as markedly reducing occurrence of relapse and oral prednisone dosage, indicating the potential privilege of our simplified regimen of low-dose rituximab in both long-term and short-term prognosis, along with sustained immunosuppression. Meanwhile, longitudinal self-control analysis in both groups also revealed continuously marked amelioration in a series of scales from baseline during at least 1 year, whereas the persistent improvement might be presented even more than 1 year in combination of rituximab. Moreover, further analysis in rituximab cohort showed no difference in any clinical outcomes between combination with single first-line and with repeated first-line treatment (≥ 2 times), while compared with delayed combination with rituximab subgroup (> 3 months), early initiation of combination (≤ 3 months) might achieve better improvements in CASE and MMSE assessments.

This study was limited by its uncontrolled design without comparison with natural course of AE or other dosing regimens, as well as retrospective observational analysis, small sample size and limited follow-up time, and a bias in selecting patients could not be completely ruled out. The data were collected during routine clinical practice rather than a formal study setting, which meant limitations of quality and quantity varied among patients. Meanwhile, we have altered AE treatment strategy when rituximab became a preferred choice for immunosuppression, causing only a small number of patients with low-dose rituximab combination enrolled for therapeutic protocol. Since randomized trials are difficult to conduct in rare diseases such as AE, real-world data might contribute to important information on treatment profiles and protocols. Although limited data on simplified regimen of combined optimal low-dose rituximab in our study, the result might be encouraging and presenting therapeutic implications for AE.

Altogether, in present study, the simplified regimen of combined low-dose rituximab (100 mg once) with common first-line therapy for AE with NSAbs, to our knowledge, firstly showed effective for short-term and long-term improvement, in parallel with reduced immunosuppressant and relapses, suggesting the advantages and benefits for combination of low-dose rituximab in the disease course. Moreover, the opportunity of rituximab protocol showed earlier initiation with better improvement, while frequency of first-line treatment had no influence on satisfactory outcome with rituximab combination. Our reports may expand therapeutic options and provide helpful references for NSAbs-associated AE, and further studies to corroborate these findings are warranted.

## Supplementary Information

**Additional file 1: Table S1.** Clinical improvements in rituximab cohort and control cohort

**Additional file 2: Table S2.** Comparison of clinical outcomes between single first-line and repeated first-line treatment subgroups in rituximab cohort

**Additional file 3: Table S3.** Comparison of clinical outcomes between early and delayed initiation of rituximab subgroups in rituximab cohort

## Data Availability

The datasets used and/or analyzed during the current study are available from the corresponding author.

## Abbreviations

AE: Autoimmune encephalitis
CNS: Central nervous system
NSAbs: Neuronal surface antibodies
NMDAR: N-Methyl-d-aspartate receptor
LGI1: Leucine-rich glioma-inactivated-1
CASPR2: Contactin-associated protein-like-2
MPPT: Methylprednisolone pulse therapy
IVIG: Intravenous immunoglobulin
PLEX: Plasma exchange
MMF: Mycophenolate mofetil
mRS: Modified Rankin Scale
CASE: Clinical Assessment Scale for Autoimmune Encephalitis
NEOS: Anti-NMDAR Encephalitis One-Year Functional Status
MMSE: Mini-mental State Examination
NPI: Neuropsychiatric Inventory
CBAs: Cell-based assays
CSF: Cerebrospinal fluid
MRI: Magnetic resonance imaging
EEG: Electroencephalogram
TLR: Toll-like receptor
BCR: B cell receptor
BAFF: B cell activating factor
ADAM: A disintegrin and metalloprotease
CD20: Cluster of differentiation 20
MHCII: Major histocompatibility complex class II
RA: Rheumatoid arthritis
NMOSD: Neuromyelitis optica spectrum disorders
MS: Multiple sclerosis
NBD: Neuro-Behçet’s disease
MG: Myasthenia gravis

## References

[1] Dalmau J, Graus F (2018). Antibody-mediated encephalitis. N Engl J Med.

[2] Dalmau J, Geis C, Graus F (2017). Autoantibodies to synaptic receptors and neuronal cell surface proteins in autoimmune diseases of the central nervous system. Physiol Rev.

[3] Dalmau J, Lancaster E, Martinez-Hernandez E (2011). Clinical experience and laboratory investigations in patients with anti-NMDAR encephalitis. Lancet Neurol.

[4] Irani SR, Alexander S, Waters P (2010). Antibodies to Kv1 potassium channel-complex proteins leucine-rich, glioma inactivated 1 protein and contactin-associated protein-2 in limbic encephalitis, Morvan’s syndrome and acquired neuromyotonia. Brain.

[5] Irani SR, Pettingill P, Kleopa KA (2012). Morvan syndrome: clinical and serological observations in 29 cases. Ann Neurol.

[6] Mader S, Brimberg L, Diamond B (2017). The role of brain-reactive autoantibodies in brain pathology and cognitive impairment. Front Immunol.

[7] Prüss H (2021). Autoantibodies in neurological disease. Nat Rev Immunol.

[8] Abboud H, Probasco JC, Irani S, Ances B, Benavides DR, Bradshaw M, Christo PP (2021). Autoimmune encephalitis: proposed best practice recommendations for diagnosis and acute management. J Neurol Neurosurg Psychiatry.

[9] Abboud H, Probasco J, Irani SR, Ances B, Benavides DR, Bradshaw M (2021). Autoimmune encephalitis: proposed recommendations for symptomatic and long-term management. J Neurol Neurosurg Psychiatry.

[10] Shin YW, Lee ST, Park KI, Jung KH, Jung KY, Lee SK, Chu K (2017). Treatment strategies for autoimmune encephalitis. Ther Adv Neurol Disord.

[11] Thaler FS, Zimmermann L, Kammermeier S, Strippel C, Ringelstein M, Kraft A (2021). Rituximab treatment and long-term outcome of patients with autoimmune encephalitis: real-world evidence from the GENERATE registry. Neurol Neuroimmunol Neuroinflamm.

[12] Lim JA, Lee ST, Moon J, Jun JS, Kim TJ, Shin YW (2019). Development of the clinical assessment scale in autoimmune encephalitis. Ann Neurol.

[13] Cai MT, Lai QL, Zheng Y, Fang GL, Qiao S, Shen CH, Zhang YX, Ding MP (2021). Validation of the clinical assessment scale for autoimmune encephalitis: a multicenter study. Neurol Ther.

[14] Zhang Y, Tu E, Yao C, Liu J, Lei Q, Lu W (2021). Validation of the clinical assessment scale in autoimmune encephalitis in Chinese patients. Front Immunol.

[15] Graus F, Titulaer MJ, Balu R (2016). A clinical approach to diagnosis of autoimmune encephalitis. Lancet Neurol.

[16] Qiao S, Wu HK, Liu LL, Zhang RR, Wang ML, Han T, Zhang SC, Liu XW (2021). Characteristics and prognosis of autoimmune encephalitis in the east of China: a multi-center study. Front Neurol.

[17] Yang CS, Yang L, Li T, Zhang DQ, Jin WN, Li MS, Su N, Zhangning N, Liu Q, Shao ZH, Yu C, Shi FD (2013). Responsiveness to reduced dosage of rituximab in Chinese patients with neuromyelitis optica. Neurology.

[18] Titulaer MJ, McCracken L, Gabilondo I, Armangué T, Glaser C, Iizuka T (2013). Treatment and prognostic factors for long-term outcome in patients with anti-NMDA receptor encephalitis: an observational cohort study. Lancet Neurol.

[19] Bien CG, Bien CI, Dogan Onugoren M, De Simoni D, Eigler V, Haensch CA (2020). Routine diagnostics for neural antibodies, clinical correlates, treatment and functional outcome. J Neurol.

[20] Lee WJ, Lee ST, Moon J, Sunwoo JS, Byun JI, Lim JA (2016). Tocilizumab in autoimmune encephalitis refractory to rituximab: an institutional cohort study. Neurotherapeutics.

[21] Lee WJ, Lee ST, Shin YW, Lee HS, Shin HR, Kim DY (2021). Teratoma removal, steroid, IVIG, rituximab and tocilizumab (T-SIRT) in anti-NMDAR encephalitis. Neurotherapeutics.

[22] Hang HL, Zhang JH, Chen DW, Lu J, Shi JP (2020). Clinical characteristics of cognitive impairment and 1-year outcome in patients with anti-LGI1 antibody encephalitis. Front Neurol.

[23] Chen W, Wang M, Gao L, Huang Z, Lin Y, Xue Q, Liu G, Zhang Y, Su Y (2021). Neurofunctional outcomes in patients with anti-leucine-rich glioma inactivated 1 encephalitis. Acta Neurol Scand.

[24] Rawlings DJ, Metzler G, Wray-Dutra M, Jackson SW (2017). Altered B cell signalling in autoimmunity. Nat Rev Immunol.

[25] Cyster JG, Allen CDC (2019). B Cell Responses: Cell Interaction Dynamics and Decisions. Cell.

[26] Nemazee D (2017). Mechanisms of central tolerance for B cells. Nat Rev Immunol.

[27] Kuraoka M, Snowden PB, Nojima T, Verkoczy L, Haynes BF, Kitamura D, Kelsoe G (2017). BCR and endosomal TLR signals synergize to increase AID expression and establish central B cell tolerance. Cell Rep.

[28] Sanderson NS, Zimmermann M, Eilinger L, Gubser C, Schaeren-Wiemers N, Lindberg RL, Dougan SK, Ploegh HL, Kappos L, Derfuss T (2017). Cocapture of cognate and bystander antigens can activate autoreactive B cells. Proc Natl Acad Sci USA.

[29] Ding H, Jian Z, Stary CM, Yi W, Xiong X (2015). Molecular pathogenesis of anti-NMDAR encephalitis. Biomed Res Int.

[30] Moscato EH, Peng X, Jain A, Parsons TD, Dalmau J, Balice-Gordon RJ (2014). Acute mechanisms underlying antibody effects in anti-N-methyl-D-aspartate receptor encephalitis. Ann Neurol.

[31] Kornau HC, Kreye J, Stumpf A, Fukata Y, Parthier D, Sammons RP, Imbrosci B, Kurpjuweit S, Kowski AB, Fukata M, Prüss H, Schmitz D (2020). Human cerebrospinal fluid monoclonal LGI1 autoantibodies increase neuronal excitability. Ann Neurol.

[32] Chen TX, Fan YT, Peng BW (2022). Distinct mechanisms underlying therapeutic potentials of CD20 in neurological and neuromuscular disease. Pharmacol Ther.

[33] Polyak MJ, Li H, Shariat N, Deans JP (2008). CD20 homo-oligomers physically associate with the B cell antigen receptor. Dissociation upon receptor engagement and recruitment of phosphoproteins and calmodulin-binding proteins. J Biol Chem.

[34] Lee DSW, Rojas OL, Gommerman JL (2021). B cell depletion therapies in autoimmune disease: advances and mechanistic insights. Nat Rev Drug Discov.

[35] Dalmau J, Gleichman AJ, Hughes EG, Rossi JE, Peng X, Lai M (2008). Anti-NMDA-receptor encephalitis: case series and analysis of the effects of antibodies. Lancet Neurol.

[36] Nosadini M, Mohammad SS, Ramanathan S, Brilot F, Dale RC (2015). Immune therapy in autoimmune encephalitis: a systematic review. Expert Rev Neurother.

[37] Chefdeville A, Honnorat J, Hampe CS, Desestret V (2016). Neuronal central nervous system syndromes probably mediated by autoantibodies. Eur J Neurosci.

[38] Bergantini L, d'Alessandro M, Cameli P, Vietri L, Vagaggini C, Perrone A, Sestini P, Frediani B, Bargagli E (2020). Effects of rituximab therapy on B cell differentiation and depletion. Clin Rheumatol.

[39] Dalakas MC (2008). Invited article: inhibition of B cell functions: implications for neurology. Neurology.

[40] Dale RC, Brilot F, Duffy LV, Twilt M, Waldman AT, Narula S (2014). Utility and safety of rituximab in pediatric autoimmune and inflammatory CNS disease. Neurology.

[41] Kaegi C, Wuest B, Schreiner J, Steiner UC, Vultaggio A, Matucci A (2019). Systematic review of safety and efficacy of rituximab in treating immune-mediated disorders. Front Immunol.

[42] Chisari CG, Sgarlata E, Arena S, Toscano S, Luca M, Patti F (2022). Rituximab for the treatment of multiple sclerosis: a review. J Neurol.

[43] Zhao C, Li C, Duan FJ, Yan Q, Zhang Z, Du Y, Zhang W (2021). Case report: repeated low-dose rituximab treatment is effective in relapsing neuro Behcet’s disease. Front Neurol.

[44] Du Y, Li C, Hao YF, Zhao C, Yan Q, Yao D, Li L, Zhang W (2022). Individualized regimen of low-dose rituximab monotherapy for new-onset AChR-positive generalized myasthenia gravis. J Neurol.

[45] van Swieten JC, Koudstaal PJ, Visser MC, Schouten HJ, van Gijn J (1988). Interobserver agreement for the assessment of handicap in stroke patients. Stroke.

