# Correction: Simplified regimen of combined low-dose rituximab for autoimmune encephalitis with neuronal surface antibodies

**DOI:** 10.1186/s12974-022-02644-2

**Published:** 2022-12-21

**Authors:** Ying Du, Chao Zhao, Juntong Liu, Chuan Li, Qi Yan, Lin Li, Yunfeng Hao, Dan Yao, Huaxing Si, Yingjun Zhao, Wei Zhang

**Affiliations:** 1 Department of Neurology, Tangdu Hospital, Fourth Military Medical University, Xi’an City, 710038 Shaanxi Province China; 2 Fujian Provincial Key Laboratory of Neurodegenerative Disease and Aging Research, Institute of Neuroscience, School of Medicine, Xiamen University, Xiamen, 361005 China

## Correction: Journal of Neuroinflammation (2022) 19:259 10.1186/s12974-022-02622-8

Following publication of the original article [1], the authors identified an error in Table 1. The “*n*” numbers in the head row of Table 1 are incorrect and inconsistent.

The correct version of Table 1 is given in this correction (Table 1).

In “Results” section under the heading “clinical outcomes”. The X in sentence “Especially, during the study…………” should be updated with 11 and 1.

It should read as,

Especially, during the study, 12 patients in total 59 patients had 12 relapses (11 with NMDAR-AE, 1 with LGI1-AE).

The original article [1] has been corrected.

**Table 1:** Characterization of the patient cohort

	TOTAL			NMDAR-AE			LGI1-AE			CASPR2-AE		
	RTX (*n* = 18)	Ctrl (*n* = 41)	*p *value	RTX (*n* = 14)	Ctrl (*n* = 27)	*p *value	RTX (*n* = 3)	Ctrl (*n* = 9)	*p *value	RTX (*n* = 1)	Ctrl (*n* = 5)	*p *value
Gender; female/male	8/10	18/23	*0.595*	7/7	11/16	*0.406*	1/2	5/4	*0.100*	0/1	2/3	*–*
Age at onset, years; mean (95% Cl)	41.88 (33–50)	37.61 (32–42)	*0.356*	35.28 (27–42)	34.22 (28–40)	*0.823*	63.33 (48–78)	52.88 (43–62)	*0.190*	70 (/)	28.40 (19–37)	*–*
From onset to diagnosis, days; median (IQR)	20 (78)	24 (52)	*0.863*	14.5 (23.75)	16 (51)	*0.185*	35 (/)	60 (63)	*0.864*	190 (/)	31 (41)	*–*
Follow-up duration, days; mean (95% Cl)	1058 (768–1349)	1362 (1136–1587)	*0.120*	1084 (802–1376)	1488 (1215–1556)	*0.059*	1086 (/)	1640 (1278)	*0.926*	244 (/)	561 (640)	*–*
Symptoms; *n* (%)												
Seizures	66.7%	61.0%	*0.455*	71.4%	66.7%	*0.100*	66.7%	55.6%	*0.100*	0.00%	40%	*–*
Cognitive impairment	94.4%	85.4%	*0.422*	92.9%	85.2%	*0.645*	100%	100%	*–*	100%	60%	*–*
Psychiatric symptoms	83.3%	87.8%	*0.690*	78.6%	88.9%	*0.393*	100%	88.9%	*0.100*	100%	80%	*–*
Decreased consciousness	44.4%	29.3%	*0.201*	57.1%	33.3%	*0.189*	0.0%	11.1%	*0.100*	0.0%	40%	*–*
Autonomic dysfunction	27.8%	22.0%	*0.742*	28.6%	25.9%	*.0.100*	33.3%	0.0%	*0.250*	0.0%	40%	*–*
Movement disorder	44.4%	26.8%	*0.151*	50%	22.2%	*0.089*	33.3%	33.3%	*0.100*	0.0%	40%	*–*
Fever	44.4%	39.0%	*0.457*	57.1%	51.9%	*0.504*	0.0%	11.1%	*0.100*	0.0%	20%	*–*
CSF/MRI/EEG profiles												
CSF cc; median, (IQR)	7 (67)	8 (23)	*0.613*	8.5 (90.5)	12 (26)	*0.264*	4 (/)	2 (4)	*0.282*	2 (/)	1 (62)	
CSF protein; mean, (95% Cl)	404.45 (318–490)	353.82 (288–419)	*0.370*	372.05 (274–469)	405.57 (313–497)	*0.637*	470.28 (93–847)	247.21 (179–314)	***0.009***	660.7 (/)	266.32 (144–387)	*–*
CSF pressure; mean, (95% Cl)	174.72 (146–203)	161.46 (140–182)	*0.463*	182.85 (147–218)	170.18 (140–199)	*0.587*	145.00 (46–243)	131.66 (100–162)	*0.632*	150 (/)	168.00 (121–214)	*–*
MRI abnormalities; *n* (%)	50.0%	56.1%	*0.440*	64.3%	51.9%	*0.336*	0.0%	77.8%	***0.045***	0.0%	40%	*–*
EEG abnormalities; *n* (%)	61.1%	43.9%	*0.175*	71.4%	48.1%	*0.137*	33.3%	33.3%	*0.100*	0.0%	40%	*–*
CSF/Serum Ab profiles												
CSF Ab positive	94.4%	80.5%	*0.252*	100%	88.9%	*0.539*	100%	77.8%	*0.100*	0.0%	40%	*–*
Serum Ab positive	38.9%	61.0%	*0.100*	28.6%	48.1%	*0.321*	66.7%	77.8%	*0.100*	100%	100%	*–*
Both Ab positive	33.3%	41.5%	*0.386*	28.6%	37.0%	*0.734*	66.7%	55.6%	*0.100*	0.0%	40%	*–*
Prior 1st-line immunotherapy; *n* (%)	100%	100%	*–*	100%	100%	*–*	100%	100%	*–*	100%	100%	*–*
1st-line therapy, *n* (%)												*–*
MPPT	72.2%	97.6%	***0.008***	85.7%	93.3%	*0.265*	33.3%	100%	***0.045***	0	100%	*–*
IVIG	94.4%	46.3%	** < ** ***.0001***	92.9%	55.6%	***0.031***	100%	33.3%	*0.182*	100%	20%	*–*
Both	66.7%	43.9%	*0.092*	78.6%	51.9%	*0.176*	33.3%	33.3%	*0.100*	0	20%	*–*
RTX therapy												
Time from RTX therapy, days; median (IQR)	74.5 (410)	*–*	*–*	128.5 (405)	*–*	*–*	6 (/)	*–*	*–*	5 (/)	*–*	*–*
No. of RTX infusions, *n*; median (IQR)	5 (4)	*–*	*–*	5 (3)	*–*	*–*	3 (/)	*–*	*–*	3 (/)	*–*	*–*
Cumulative RTX dose, *g*; median (IQR)	500 (400)	*–*	*–*	500 (300)	*–*	*–*	300 (/)	*–*	*–*	300 (/)	*–*	*–*
1st to last infusion, days; median (IQR)	389 (864)	*–*	*–*	457.5 (863)	*–*	*–*	15 (/)	*–*	*–*	214 (/)	*–*	*–*
Averaged dose of prednisone, mg/day; median (IQR)	4.375 (7.67)	27.13 (21.67)	** < ** ***.0001***	5.563 (4.011)	28.89 (21.67)	** < ** ***.0001***	0	10.83 (19.52)	***0.009***	0	25.71 (20.057)	*–*
Relapses; *n* (%)												
After 1st-line therapy	33.3%	12.2%	*0.074*	42.9%	18.5%	*0.140*	33.3%	0.0%	*0.250*	0	0	*–*
After RTX therapy	0	*–*	*–*	0	*–*	*–*	0	*–*	*–*	0	*–*	*–*

NOTE: *p* values <0.05 are considered to be statistically significant, and are indicated in italic and bolditalic

*AE* autoimmune encephalitis, *NMDAR* *N*-methyl-d-aspartate receptor, *LGI1* leucine-rich glioma-inactivated-1, *CASPR2* contactin-associated protein-like-2, *RTX* rituximab, *Ctrl* control, *Cl* confidence interval, *IQR* interquartile rang, *CSF* cerebrospinal fluid, *MRI* magnetic resonance imaging, *EEG* electroencephalogram, *cc* cell count, *Ab* antibody, *MPTP* methylprednisolone pulse therapy, *IVIG* intravenous immunoglobulin.

## References

[1] Du Y, Zhao C, Liu J, Li C, Yan Q, Li L, Hao Y, Yao D, Si H, Zhao Y, Zhang W (2022). Simplified regimen of combined low-dose rituximab for autoimmune encephalitis with neuronal surface antibodies. J Neuroinflammation.

