# Supplementary material for: Simplified regimen of combined low-dose rituximab for autoimmune encephalitis with neuronal surface antibodies
Source: J Neuroinflammation. 2022 Oct 22;19:259. doi: 10.1186/s12974-022-02622-8 (PMC9587594; doi:10.1186/s12974-022-02622-8)
Supplement: Supplementary file 1 — Additional file 1: Table S1. Clinical improvements in rituximab cohort and control cohort [file 12974_2022_2622_MOESM1_ESM.docx]

Supplementary Table 1. Clinical improvements in rituximab cohort and control cohort

| **Therapy** | **Evaluation Scales** | **Baseline** | **1st visit** |  | **2nd visit** |  | **3rd visit** |  | **last visit** |  |
| --- | --- | --- | --- | --- | --- | --- | --- | --- | --- | --- |
|  |  | **Median (IQR)** | **± Median (IQR)** | ***p*** | **± Median (IQR)** | ***p*** | **± Median (IQR)** | ***p*** | **± Median (IQR)** | ***p*** |

# Rituximab cohort (n=18)

| CASE | 8(7.25) | -5(4.5) | ***.003*** | -6.5(5.5) | ***<.0001*** | -6.5(7.3) | ***<.0001*** | -7(7) | ***<.0001*** |
| --- | --- | --- | --- | --- | --- | --- | --- | --- | --- |
| MRs | 4(1.25) | -1(1) | ***.007*** | -2(2) | ***<.0001*** | -3(2) | ***<.0001*** | -3.5(2) | ***<.0001*** |
| MMSE | 14.5(16.25) | +8(8.8) | ***.005*** | +11(15.5) | ***<.0001*** | +10.5(16) | ***<.0001*** | +13(15.3) | ***<.0001*** |
| Patient NPI | 15(20.5) | -14(14.3) | ***.001*** | -13(16) | ***<.0001*** | -14(20) | ***<.0001*** | -15(20.8) | ***<.0001*** |
| Caregiver NPI | 6.5(5.75) | -6(5) | ***.001*** | -5(7.3) | ***<.0001*** | -6.5(7.8) | ***<.0001*** | -6.5(6.3) | ***<.0001*** |
| CASE | 6(2) | -3(2) | ***<.0001*** | -3(3) | ***<.0001*** | -4(3) | ***<.0001*** | -4(3) | ***<.0001*** |
| MRs | 4(1) | -1(0.5) | ***.015*** | -2(2) | ***<.0001*** | -2(1) | ***<.0001*** | -3(1) | ***<.0001*** |
| MMSE | 20(15.5) | +3(7.5) | ***.092*** | +4(13) | ***.001*** | +5(14) | ***<.0001*** | +7(14) | ***<.0001*** |
| Patient NPI | 9(10) | -6(10) | ***.001*** | -7(11.5) | ***<.0001*** | -8(11.5) | ***<.0001*** | -8(11.5) | ***<.0001*** |
| Caregiver NPI | 5(5) | -2(4) | ***.001*** | -3(5.5) | ***<.0001*** | -4(5) | ***<.0001*** | -3(5) | ***<.0001*** |

**Control cohort (n=41)**

Detailed clinical status was evaluated by a series of AE-associated scales at baseline before treatment and continuous 4 visits after treatment. 1st visit: at discharge, 2nd visit: 6 months later, 3rd visit: 12 months later, 4th visit: last follow-up with at least >12 months.

CASE=the Clinical Assessment Scale for Autoimmune Encephalitis; mRS=the modified Rankin Scale score; MMSE= the Mini-mental State Examination score; NPI=the Neuropsychiatric Inventory; IQR=interquartile rang. p Values reaching statistical significance are indicated in bold.
