# Supplementary material for: Simplified regimen of combined low-dose rituximab for autoimmune encephalitis with neuronal surface antibodies
Source: J Neuroinflammation. 2022 Oct 22;19:259. doi: 10.1186/s12974-022-02622-8 (PMC9587594; doi:10.1186/s12974-022-02622-8)
Supplement: Supplementary file 2 — Additional file 2: Table S2. Comparison of clinical outcomes between single first-line and repeated first-line treatment subgroups in rituximab cohort [file 12974_2022_2622_MOESM2_ESM.docx]

Supplementary Table 2. Comparation of clinical outcomes between single first-line and repeated first-line treatment subgroups in rituximab cohort

|  |  | **Baseline** |  |  | **1st visit** |  |  | **2nd visit** |  |  | **3rd visit** |  |  | **last visit** |  |  |
| --- | --- | --- | --- | --- | --- | --- | --- | --- | --- | --- | --- | --- | --- | --- | --- | --- |
| **Evaluation Scales** | | **Single (n=9)** | **Repeat (n=9)** |  | **Single (n=9)** | **Repeat (n=9)** |  | **Single (n=9)** | **Repeat (n=9)** |  | **Single (n=9)** | **Repeat (n=9)** |  | **Single (n=9)** | **Repeat (n=9)** |  |
|  |  |  |  | ***p*** |  |  | ***p*** |  |  | ***p*** |  |  | ***p*** |  |  | ***p*** |
| **CASE** | Scores median(IQR) | 9(5.5) | 6(8.5) | *.546* | 3(4) | 3(5) | *.730* | 2(2) | 1(5) | *.790* | 2(1) | 1(2.5) | *.387* | 1(2) | 0(1) | *.387* |
| **MRs** | Scores median(IQR) | 4(2) | 4(0) | *.730* | 2(1) | 2(1.5) | *.666* | 2(1) | 2(2.5) | *.100* | 1(1) | 1(2) | *.297* | 1(1) | 0(1.5) | *.387* |
| **MMSE** | Scores median(IQR) | 12(17.5) | 16(5) | *.190* | 22(9) | 24(10) | *.340* | 24(7) | 28(8) | *.222* | 26(7) | 28(6) | *.387* | 27(2.5) | 29(2) | *.161* |
| **Patient NPI** | Scores median(IQR) | 16(20.5) | 14(19.5) | *.666* | 0(5.5) | 2(5) | *.730* | 2(4) | 0(4) | *.546* | 0(2) | 0(1.5) | *.100* | 0(2) | 0(0) | *.666* |
| **Caregiver NPI** | Scores median(IQR) | 8(5.5) | 6(8) | *.489* | 0(3.5) | 1(2) | *.100* | 1(2) | 0(1) | *.340* | 0(0) | 0(1) | *.489* | 0(1) | 0(0) | *.489* |
| Detailed clinical status was evaluated by a series of AE-associated scales at baseline before treatment and continuous 4 visits after the first rituximab infusion. 1st visit: at discharge, 2nd visit: 6 months later, 3rd visit: 12 months later, 4th visit: last follow-up with at least >12 months.  Single=single fist-line treatment subgroup in rituximab cohort; Repeat=repeat fist-line treatment subgroup in rituximab cohort; CASE=the Clinical Assessment Scale for Autoimmune Encephalitis; MRs=the modified Rankin Scale score; MMSE=the Mini-mental State Examination score; NPI=the Neuropsychiatric Inventory; IQR=interquartile rang. p Values reaching statistical significance are indicated in bold. | | | | | | | | | | | | | | | | |
