# Supplementary material for: Simplified regimen of combined low-dose rituximab for autoimmune encephalitis with neuronal surface antibodies
Source: J Neuroinflammation. 2022 Oct 22;19:259. doi: 10.1186/s12974-022-02622-8 (PMC9587594; doi:10.1186/s12974-022-02622-8)
Supplement: Supplementary file 3 — Additional file 3: Table S3. Comparison of clinical outcomes between early and delayed initiation of rituximab subgroups in rituximab cohort [file 12974_2022_2622_MOESM3_ESM.docx]

Supplementary Table 3. Comparation of clinical outcomes between early and delayed initiation of rituximab subgroups in rituximab cohort

|  |  | **Baseline** |  |  | **1st visit** |  |  | **2nd visit** |  |  | **3rd visit** |  |  | **last visit** |  |  |
| --- | --- | --- | --- | --- | --- | --- | --- | --- | --- | --- | --- | --- | --- | --- | --- | --- |
| **Evaluation Scales** | | **Early (n=10)** | **Delayed (n=8)** |  | **Early (n=10)** | **Delayed (n=8)** |  | **Early (n=10)** | **Delayed (n=8)** |  | **Early (n=10)** | **Delayed (n=8)** |  | **Early (n=10)** | **Delayed (n=8)** |  |
|  |  |  |  | ***p*** |  |  | ***p*** |  |  | ***p*** |  |  | ***p*** |  |  | ***p*** |
| **CASE** | Scores median(IQR) | 7(10.5) | 8.5(5.75) | *.100* | 2(6.25) | 3.5(3.25) | *.573* | 2(4.25) | 2(2.5) | *.897* | 1(2.25) | 2(0.75) | *.122* | 0(0.25) | 1(1.75) | ***.039*** |
| **MRs** | Scores median(IQR) | 4(0.5) | 4(1.75) | *.780* | 2.5(1.25) | 2(0.75) | *.460* | 2(1.5) | 1.5(1) | *.633* | 1(2) | 1(1) | *.573* | 0(1.25) | 1(0.75) | *.203* |
| **MMSE** | Scores median(IQR) | 15(16.5) | 13(17.5) | *.400* | 24(6) | 21.5(11) | *.573* | 27(6.5) | 23.5(8.75) | *.573* | 28.5(5.5) | 26(6.25) | *.203* | 29(1) | 26.5(1.75) | ***.046*** |
| **Patient NPI** | Scores median(IQR) | 16(13.25) | 12.5(25.5) | *.842* | 2.5(4.5) | 0(6.25) | *.633* | 0(4) | 1(4) | *.829* | 0(1.25) | 0(2) | *.762* | 0(0) | 0(3) | *.573* |
| **Caregiver NPI** | Scores median(IQR) | 6.5(5.75) | 7(8.5) | *.905* | 1.5(2.25) | 0(2.75) | *.696* | 0(1.25) | 0.5(2) | *.633* | 0(1) | 0(0) | *.573* | 0(0) | 0(1) | *.408* |
| Detailed clinical status was evaluated by a series of AE-associated scales at baseline before treatment and continuous 4 visits after the first rituximab infusion. 1st visit: at discharge, 2nd visit: 6 months later, 3rd visit: 12 months later, 4th visit: last follow-up with at least >12 months.  Early=early initiation of rituximab treatment subgroup in rituximab cohort; Delayed=delayed initiation of rituximab treatment subgroup in rituximab cohort; CASE=the Clinical Assessment Scale for Autoimmune Encephalitis; MRs=the modified Rankin Scale score; MMSE=the Mini-mental State Examination score; NPI=the Neuropsychiatric Inventory; IQR=interquartile rang. p Values reaching statistical significance are indicated in bold. | | | | | | | | | | | | | | | | |
